# Optimizing prognosis-related key miRNA-target interactions responsible for cancer metastasis

**DOI:** 10.18632/oncotarget.22724

**Published:** 2017-11-27

**Authors:** Hongying Zhao, Huating Yuan, Jing Hu, Chaohan Xu, Gaoming Liao, Wenkang Yin, Liwen Xu, Li Wang, Xinxin Zhang, Aiai Shi, Jing Li, Yun Xiao

**Affiliations:** ^1^ College of Bioinformatics Science and Technology, Harbin Medical University, Harbin 150081, China; ^2^ Department of Ultrasonic Medicine, The 1st Affiliated Hospital of Heilongjiang University of Chinese Medicine, Harbin 150040, China

**Keywords:** miRNA, key miRNA-target interactions, prognosis, cancer metastasis, human cancers

## Abstract

Increasing evidence suggests that the abnormality of microRNAs (miRNAs) and their downstream targets is frequently implicated in the pathogenesis of human cancers, however, the clinical benefit of causal miRNA-target interactions has been seldom studied. Here, we proposed a computational method to optimize prognosis-related key miRNA-target interactions by combining transcriptome and clinical data from thousands of TCGA tumors across 16 cancer types. We obtained a total of 1,956 prognosis-related key miRNA-target interactions between 112 miRNAs and 1,443 their targets. Interestingly, these key target genes are specifically involved in tumor progression-related functions, such as ‘cell adhesion’ and ‘cell migration’. Furthermore, they are most significantly correlated with ‘tissue invasion and metastasis’, a hallmark of metastasis, in ten distinct types of cancer through the hallmark analysis. These results implicated that the prognosis-related key miRNA-target interactions were highly associated with cancer metastasis. Finally, we observed that the combination of these key miRNA-target interactions allowed to distinguish patients with good prognosis from those with poor prognosis both in most TCGA cancer types and independent validation sets, highlighting their roles in cancer metastasis. We provided a user-friendly database named miRNATarget (freely available at http://biocc.hrbmu.edu.cn/miRNATar/), which provides an overview of the prognosis-related key miRNA-target interactions across 16 cancer types.

## INTRODUCTION

MicroRNAs (miRNAs) are a class of small non-protein-coding RNAs [1]. MiRNAs, as important regulators of tumorigenesis [2, 3], are involved in many cancer-related processes such as cell apoptosis, proliferation and metastasis. For example, miR-7 regulates glioblastoma (GBM) cell invasion by targeting focal adhesion kinase [4]. Furthermore, these miRNAs may contribute to tumor progression primarily through inhibiting the expression of some key downstream target genes [4–6]. MiR-155 reduces the aggressiveness of tumor cell dissemination by directly suppressing the expression of *TCF4* which is a regulator of epithelial-to-mesenchymal transition (EMT) [5]. MiR-155 can regulate the proliferation and invasion of clear cell renal cell carcinoma cells by targeting *E2F2* [6]. It underscores the important role of key miRNA-target interactions in the molecular mechanisms of how dysfunctional miRNAs are involved in cancer prognosis [7].

Also, advances in small non-coding RNA transcriptome have generated many candidate miRNA markers with potential clinical value in diverse malignancies [8–11]. For example, miR-1 acts as a prognostic marker in prostate cancer by inhibiting cell proliferation and motility [11]. However, clinical benefit of the causal miRNA-target interactions remains poorly characterized. Some progress in this area has been made, for example, epigenetic silencing of the tumor suppressor miR-124a confers a poor prognosis in acute lymphoblastic leukemia by regulating *CDK6* expression [12]. The importance of these miRNA-target interactions in cancer prognosis is widely accepted, but a systematic approach to identify prognosis-related miRNA-target interactions is lacking.

In this study, we propose a computational method to optimize prognosis-related key miRNA-target interactions by integrating miRNA, mRNA expression profiles and clinical information. We applied our method to 16 TCGA cancer types and identified a total of 1,956 prognosis-related key miRNA-target interactions consisting of 112 miRNAs and 1,443 target genes. We found that these prognosis-related key miRNA-target interactions were specifically involved in tumor progression-related functions, such as ‘cell adhesion’ and ‘cell migration’. Hallmark analysis revealed that a hallmark of metastasis ‘tissue invasion and metastasis’ was most significantly influenced by key target genes in ten distinct types of cancer. In most TCGA cancer types, the combination of key miRNA-target interactions could act as an independent cancer-specific signature associated with overall survival. We provided a free online database named miRNATarget for optimizing prognosis-related key miRNA-target interactions across 16 types of cancer.

## RESULT

### Optimizing prognosis-related key miRNA-target interactions

**Figure 1:**
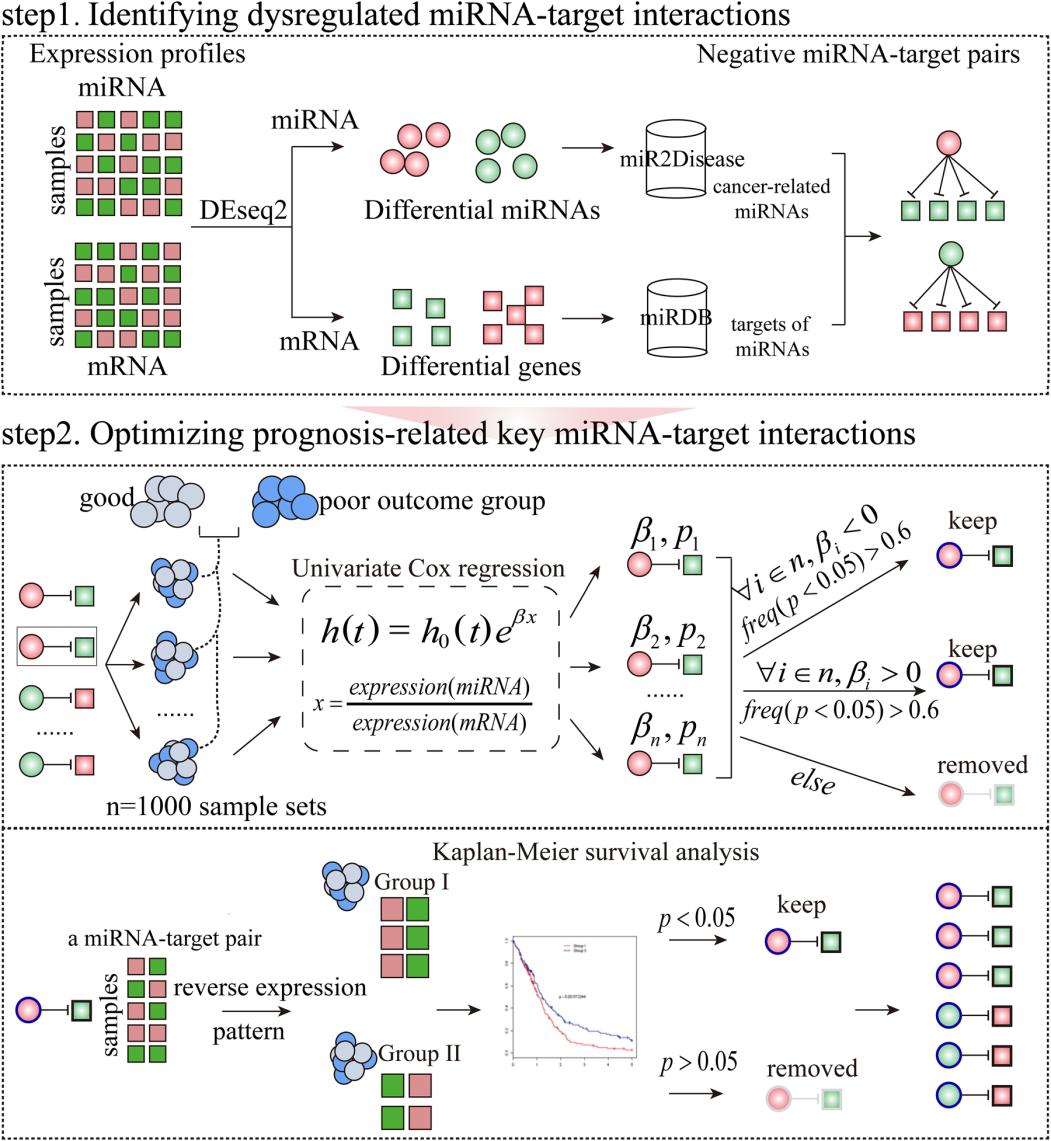
The workflow to identify prognosis-related key miRNA-target interactions in a specific condition Step 1: Identifying negative miRNA-target interactions between differentially expressed miRNAs in miR2Disease and differentially expressed mRNAs. Step 2: Optimizing miRNA-target interactions which are correlated with survival using Cox proportional hazard regression model. Furthermore, for a miRNA-target interaction, patients with specific cancer types were divided into two subgroups on the basis of reverse expression pattern of the miRNA and its target. Finally, a miRNA-target interaction with log-rank test *P*-value<0.05 was considered as a prognosis-related key miRNA-target interaction (see Method section for details). The coloured circles and squares represent the differentially expressed miRNAs and genes, respectively. Red symbols correspond to upregulation, whereas green symbols indicate downregulation.

Given that miRNAs are associated with cancer prognosis and key miRNA targets are functionally important in cancer prognosis, we asked whether the causal miRNA-target interactions could serve as prognostic indicators in human cancers. To address this question, we selected 5,353 patients involving 16 different cancer types that had expression of miRNAs and mRNAs and survival data from TCGA (Table 1). We developed a method and applied it to the 16 TCGA cancer types to identify prognosis-related key miRNA-target interactions (Figure 1, see Method section for further details).

**Table 1:** The detail information of patients in 16 cancer types

Cancer type	Gene expression technique	Gene expression tumor samples	Gene expression normal samples	miRNA expression technique	miRNA expression tumor samples	miRNA expression normal samples	Clinical data samples
BLCA	RNA-seq	241	19	miRNA-seq	252	19	195
BRCA	RNA-seq	1095	113	miRNA-seq	755	87	734
CESC	RNA-seq	304	3	miRNA-seq	307	3	299
COAD	RNA-seq	261	41	miRNA-seq	469	8	231
ESCA	RNA-seq	184	11	miRNA-seq	186	13	182
GBM	microarry	395	10	microarry	436	10	372
HNSC	RNA-seq	450	43	miRNA-seq	419	43	405
KIRC	RNA-seq	518	72	miRNA-seq	236	71	219
KIRP	RNA-seq	172	30	miRNA-seq	198	32	153
LIHC	RNA-seq	371	50	miRNA-seq	372	50	357
LUAD	RNA-seq	488	58	miRNA-seq	434	46	368
LUSC	RNA-seq	490	50	miRNA-seq	331	45	239
OV	microarry	568	8	microarry	568	8	555
PAAD	RNA-seq	178	4	miRNA-seq	178	4	178
STAD	RNA-seq	415	35	miRNA-seq	399	41	364
THCA	RNA-seq	505	59	miRNA-seq	506	59	502

**Figure 2:**
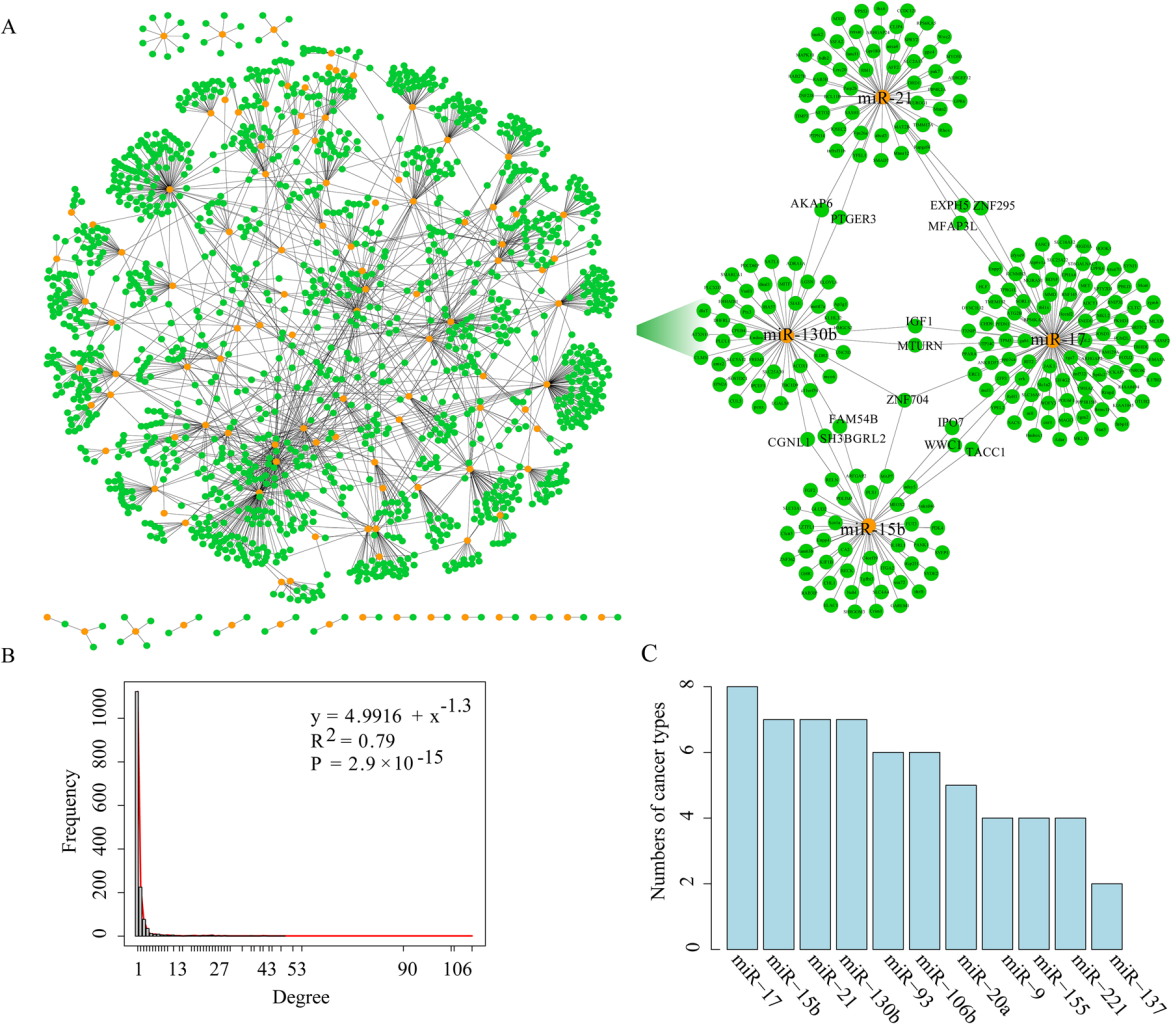
The layout of the miRNA-target network and its structure features **(A).** The left panel shows the global miRNA-target interaction network, and the right panel shows a sub-network including four miRNAs (miR-17, miR-15b, miR-21 and miR-130b) and their key target genes. Orange nodes mark miRNAs and green nodes represent their target genes. An edge represents a negative regulation from miRNA to one of its targets. **(B).** The degree distribution of the miRNA-target interaction network. **(C).** The graph indicating the number of cancer types in which the hub miRNA is detected.

As a result, we obtained a total of 1,956 prognosis-related key miRNA-target interactions between 112 miRNAs and 1,443 target genes. The size of the prognosis-related key miRNA-target interactions ranged from 3 to 580, with an average of 124 interactions per cancer type (Table 2 and Figure 2A). As an example, 528 prognosis-related key miRNA-target interactions involving 51 miRNAs and 467 target genes were observed in GBM. As illustrated by two key miRNA-target interactions, miR-155:*MXI1* (*P*-value=0.001, log-rank test, Supplementary Figure 1A) and miR-21:*DRD1* (*P*-value=0.006, Supplementary Figure 1B), it seems that key regulatory pairs are responsible for clinical prognosis. In fact, previous studies have reported that miR-155, a GBM progression-related miRNA [13], can promote glioma cell proliferation by regulating *MXI1* [14, 15]. MiR-21 can promote glioma invasion [16], and its target *DRD1* is related to cancer metastasis [17]. In KIRC, the key miRNA-target interactions, such as miR-29a:*EDNRB* and miR-17:*MFAP3L*, are highly predictive of clinical outcome (*P*-value<0.05, Supplementary Figure 1C and Supplementary Figure 1D). Based on these interactions, a prognosis-related key miRNA-target network was constructed (Figure 2A). Next, the degree distribution of the prognosis-related key miRNA-target network follows a power-law distribution with a slope of −1.3 and R^2^=0.79 (*P*-value=2.9e-15, Figure 2B), implying that the network is not random but is characterized by a core set of organizing principles in its structure that distinguishes it from randomly generated networks [18].

**Table 2:** Prognosis-related key miRNA-target interactions in 16 cancer types

Cancer type	BLCA	BRCA	CESC	CO AD	ES CA	GBM	HNSC	KI RC	KI RP	LIHC	LU AD	LUSC	OV	PA AD	STAD	THCA
miRNA-target	203	93	31	4	12	528	115	580	181	71	51	6	84	3	7	10
miRNA	21	23	17	3	9	51	9	65	34	17	19	6	37	2	4	8
Targets	109	85	23	3	10	467	114	424	163	65	41	6	59	3	7	9

Hubs are of general interest as they represent the most influential components of a network and, accordingly, tend to be essential. Hub miRNAs are commonly defined as the top 10% of the nodes by degree [19] and regulate ≥10 target genes. The analysis identified a total of 11 hub miRNAs including miR-20a, miR-221, miR-17, miR-137, miR-21, miR-130b, miR-15b, miR-9, miR-106b, miR-93 and miR-155 shared by at least two cancer types (Figure 2C). The top hub miRNAs (such as miR-17, miR-21, miR-130b and miR-15b) have been reported to be associated with tumor cell migration, invasion and metastasis (such as in glioma, breast cancer, ovarian carcinoma and endometrial cancer) [16, 20–23]. A sub-network composed of the top four hub miRNAs and their 224 key target genes was shown in Figure 2A (right box). There are 14 genes (including *ZNF704*, *AKAP6*, *EXPH5*, *MFAP3L*, *MTURN*, *IPO7*, *TACC1*, *PTGER3*, *ZNF296*, *IGF1*, *WWC1*, *CGNL1*, *SH3BGRL2* and *FAM54B*) regulated by at least two miRNAs in the sub-network, many of which are involved in tumor progression and prognosis [24–29]. For example, activation of *MFAP3L* can promote colorectal cancer cell invasion and metastasis [30]. The gene *TACC1* is associated with endocrine therapy resistance in breast cancer [24]. Insulin-like growth factor-1 (*IGF1*) is correlated with proliferation and migration of hepatocellular carcinoma [29].

### Prognosis-related key miRNA-target interactions are responsible for cancer metastasis

#### GO term enrichment analysis reveals the association between prognosis-related key miRNA-target interactions and tumor progression-related functions

**Figure 3:**
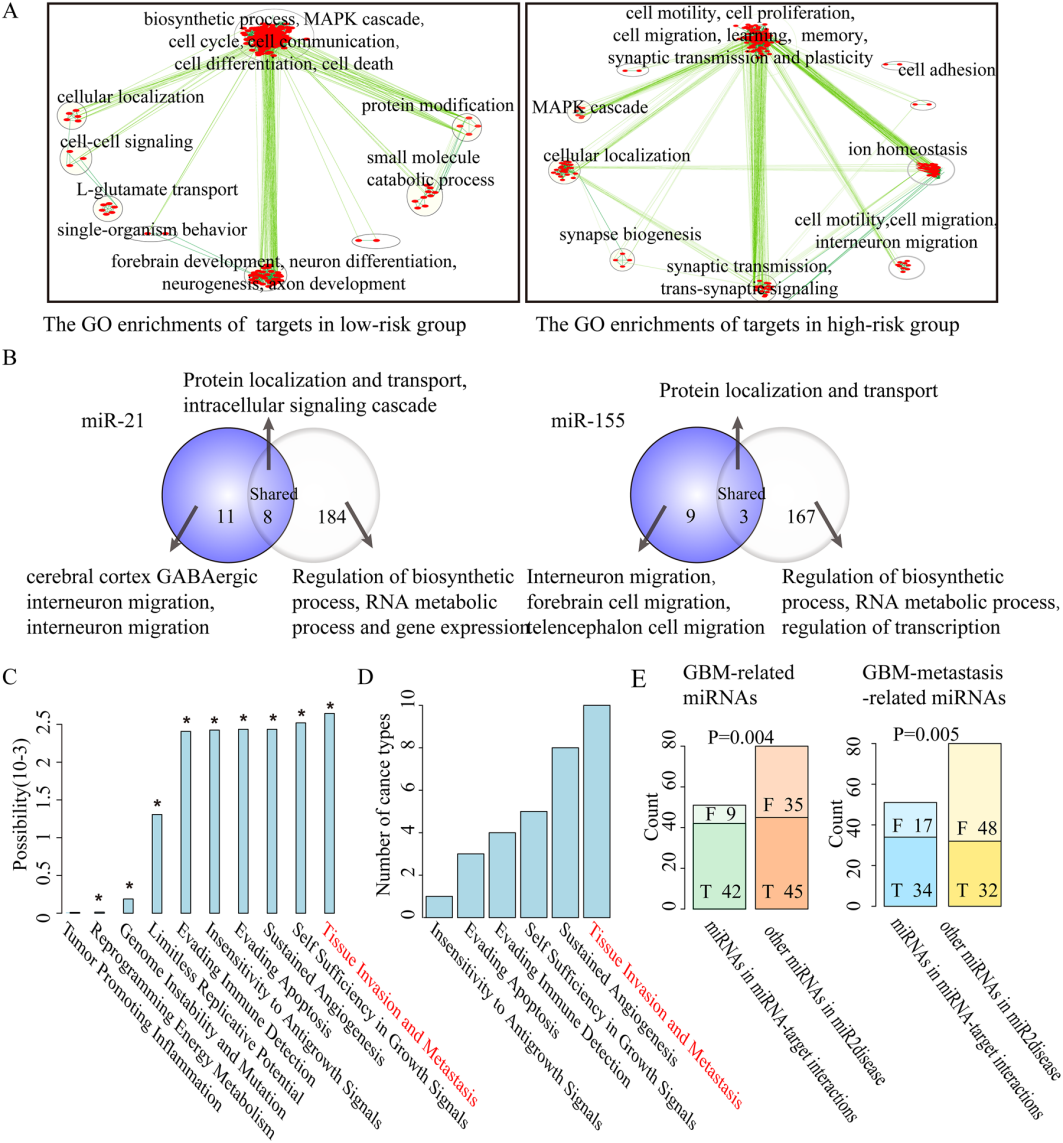
The function explorations of prognosis-related key miRNA-target interactions selected from GBM **(A).** Map of enriched functions for genes in prognosis-related key miRNA-target interactions based on DAVID output. **(B).** Overlap between the DAVID output detected by key targets (left, purple) and all targets (right) of miR-21 and miR-155 in GBM. The labels of top most significant GO terms are showed. **(C).** The impact of prognosis-related key miRNA-target interactions on 10 hallmarks of cancer. Asterisks represent significant levels at *P*-value<0.05 based on permutation tests. **(D).** A hallmark of metastasis ‘tissue invasion and metastasis’ as most frequently significantly influenced by key miRNA targets across the 16 cancer types are sorted vertically according to the number of cancer types. **(E).** Bar graphs showing the number of selected miRNAs and other cancer-related miRNAs classified into GBM-related miRNAs (left panel), GBM metastasis-related miRNAs (right panel) or not, respectively.

To explore the function of prognosis-related key miRNA-target interactions in cancer, we investigated the biological functions of their key targets. In a Cox regression model, a positive regression coefficient means a high risk of recurrence as expression ratio of miRNA-target increases, whereas a negative coefficient indicates the opposite effect. We thus categorized key miRNA-target interactions into two groups according to the regression coefficients derived from the Cox regression analysis: the high-risk group with positive regression coefficients and the low-risk group with negative regression coefficients. To uncover the biological function of key miRNA-target interactions, we performed Gene Ontology (GO) enrichment analysis for key targets of miRNAs in the high-risk and low-risk group using DAVID [31] with a false discovery rate (FDR) of 0.05 in each cancer type, respectively. Take GBM for example, GO enrichment analysis revealed that key targets of miRNAs in the low-risk group were mostly enriched in ‘cell cycle’, ‘cell communication’ and ‘MAPK cascade’, those in the high-risk group are mostly enriched in ‘cell adhesion’, ‘cell motility’, ‘interneuron migration’ and ‘synaptic plasticity’ (Figure 3A). While in KIRC, key targets of miRNAs in the low-risk group were mostly enriched in ‘cell death’, ‘cell proliferation’ and ‘vasculogenesis’, those in the high-risk group were mostly enriched in ‘cell adhesion’, ‘cell motion’ and ‘epithelial cell migration’ (Supplementary Figure 2A), which is consistent with that KIRC is a typical metabolic disease [32].

We further used GO enrichment analysis to check whether key targets of miRNAs might be biased toward particular biological functions relative to their predicted targets. In GBM and KIRC, a set of distinct biological functions were found to be enriched by key targets of miRNAs. For instance, except for some common functions such as ‘protein localization and transport’, key targets of miR-21 in GBM were specifically related to ‘cerebral cortex GABAergic interneuron migration’, ‘interneuron migration from the subpallium to the cortex’ and ‘substrate-independent telencephalic tangential interneuron migration’. Key targets of miR-155 in GBM were specifically related to ‘interneuron migration from the subpallium to the cortex’, ‘cerebral cortex GABAergic interneuron migration’ and ‘telencephalon and forebrain cell migration’ (Figure 3B). Previous studies reported that the active migration of tumor cells is crucial for cancer metastasis and progression [33]. It was consistent that oncogenic miR-21 and miR-155 involved in the progression and invasion of GBM [16, 34], and a set of their common key targets such as *LHX6* [35], *DRD1* [17], *NEUROG1* [36] and *RAB27B* [37] were also associated with tumor progression. Similarly, in KIRC we observed that key targets of miR-15b were specifically enriched in ‘regulation of epithelial cell migration’, ‘response to hormone stimulus’ and ‘tissue morphogenesis’. Key targets of miR-204 in KIRC were specifically enriched in ‘apoptosis’ and ‘programmed cell death’ (Supplementary Figure 2B), consistent with inhibition of renal clear cell carcinoma tumor growth [38].

#### Hallmark analysis reveals a significant influence of prognosis-related key miRNA-target interactions on cancer metastasis

Furthermore, hallmarks of cancer were proposed that cancer cells acquire a number of biological characteristics during the initiation and progression of tumors [39]. We used these hallmarks to investigate whether key target genes of miRNAs are associated with biological capabilities of tumor cells. Hallmark-associated KEGG pathways were identified and random walk–with–restart algorithm over a protein interaction network was used to estimate the impact of prognosis-related key miRNA targets on hallmark-associated KEGG pathways (for details, see Methods) for each cancer type. We performed 1,000 random permutations to calculate the statistical significance of the association between prognosis-related key miRNA-target interactions and a specific cancer hallmark. In GBM, the top three hallmarks of cancer significantly influenced by key miRNA targets are ‘tissue invasion and metastasis’, ‘self sufficiency in growth signals’, and ‘sustained angiogenesis’ (Figure 3C). Analogously, in KIRC, the top three hallmarks of cancer are ‘sustained angiogenesis’, ‘tissue invasion and metastasis’ and ‘insensitivity to antigrowth signal’ (Supplementary Figure 2C). It is of interest to note that a hallmark of metastasis ‘tissue invasion and metastasis’ is significantly influenced by prognosis-related key miRNA-target interactions in ten distinct types of cancer (Figure 3D, Supplementary Figure 3). These findings implicate that the prognosis-related key miRNA-target interactions are associated with cancer metastasis.

#### A survey of the published literature supports the association of prognosis-related key miRNA-target interactions with cancer metastasis

On the basis of these results, we further verified the association between prognosis-related key miRNA-target interactions and cancer metastasis. By searching the PubMed database, we found that key miRNAs (42 out of 51; 82.4%) in GBM were significantly associated with the risk of GBM when compared with other disease-associated miRNAs (45 out of 80; 56.3%) from miR2Disease (*P*-value=0.004, chi-square test). Importantly, we confirmed that key miRNAs (34 out of 51; 66.7%) were significantly associated with metastasis of GBM when compared with other disease-associated miRNAs (32 out of 80; 40.0%; *P*-value=0.005, chi-square test) (Figure 3E). It may suggest the important role of key miRNAs in cancer metastasis, since similar results were also observed in KIRC (Supplementary Figure 2D).

#### The clinical benefit of the combination of key miRNA-target interactions supports their function in cancer metastasis

**Figure 4:**
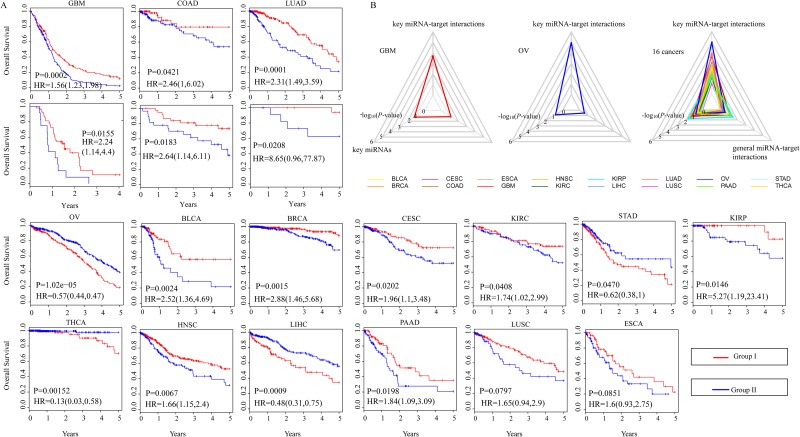
Clinical significance of the combination of key miRNA-target interactions for 16 human cancers **(A).** K-means clustering of patients with a given cancer type, according to expression ratios of miRNAs to their target genes is performed. The Kaplan-Meier plots are used to visualize the survival probabilities for two groups of patients. The differences between the two curves are determined by log-rank test. GBM, COAD and LUAD have an additional validation set. **(B).** Radar diagrams are used to visualize the –log 10-transformation *P*-values from log-rank tests of key miRNA-target interactions, key miRNAs and predicted miRNA-target interactions in GBM, OV and 16 cancer types.

We next hypothesized that cancer metastasis is associated with poor prognosis [41–43]. We investigated the impact of the combination of these key miRNA-target interactions on cancer prognosis in support of their function in cancer metastasis. As an example, in OV, 84 prognosis-related key miRNA-target interactions involving 37 miRNAs and 59 targets were used to cluster 555 OV patients into two groups on the basis of expression ratios of miRNAs to their targets using k-means clustering. We observed that the combination of key miRNA-target interactions allowed to distinguish OV patients with good prognosis from those with poor prognosis (*P*-value=1.02e^-5^, log-rank test, Figure 4A). Repeating this process for each cancer type, we observed that the combination of key miRNA-target interactions could be highly predictive of clinical outcome in most TCGA cancer types, except for LUSC and ESCA (Figure 4A). Additionally, three additional data sets containing mRNA expression, miRNA expression and clinical information of 60 GBM samples (CGCA), 65 COAD samples (GSE29623) and 32 LUAD samples (GSE63805 and GSE63459) were used to further confirm the clinical benefit of key miRNA-target interactions. A significant difference was observed in overall survival between two groups of patients (*P*-value=0.02 for GBM with a hazard ratio of 2.24, *P*-value=0.02 for COAD with a hazard ratio of 2.64 and *P*-value=0.02 for LUAD with a hazard ratio of 8.65, log-rank test, Figure 4A), which showed comparable performance on the validation set of independent tumors. Moreover, patients with short progression-free survival (PFS) or with distant metastasis (TNM stage) or with poor functional status (karnofsky performance status score (KPS) <60) are more likely to undergo cancer metastasis. Thus, we divided 372 GBM patients into two groups according to expression ratio of miRNAs to their key targets and performed significance analysis. The results showed significant difference between two groups in terms of PFS (*P*-value=0.016, log-rank test, Supplementary Figure 4A) and KPS (*P*-value=0.024, Fisher’s exact test, Supplementary Figure 4B). The percentage of GBM patients with (M1) or without (M0) distant metastases showed extremely close to significance (*P*-value=0.077, Fisher’s exact test, Supplementary Figure 4C).

Additionally, in 93.8% (15 out of 16) of cancer types, key miRNA-target interactions could improve the significance of between-group survival differences relative to miRNAs alone and general miRNA-target interactions (Figure 4B). As in GBM and OV, we found that miRNAs alone or general miRNA-target interactions could not help to distinguish patients with good prognosis from those with poor prognosis (*P*-value>0.05, log-rank test, Figure 4B). Furthermore, for each type of cancer, a multivariable Cox proportional hazards regression model was used to assess the association between the combination of key miRNA-target interactions and overall survival after adjusting for appropriate covariates: gender and age. Most cancer types (11 out of 16; 68.8%) showed that key miRNA-target interactions remained an independent prognostic signature for survival (Supplementary Figure 4D). These results highlight the important roles of prognosis-related key miRNA-target interactions in cancer metastasis.

### MiRNAs regulate their key targets in a cancer-specific manner

**Figure 5:**
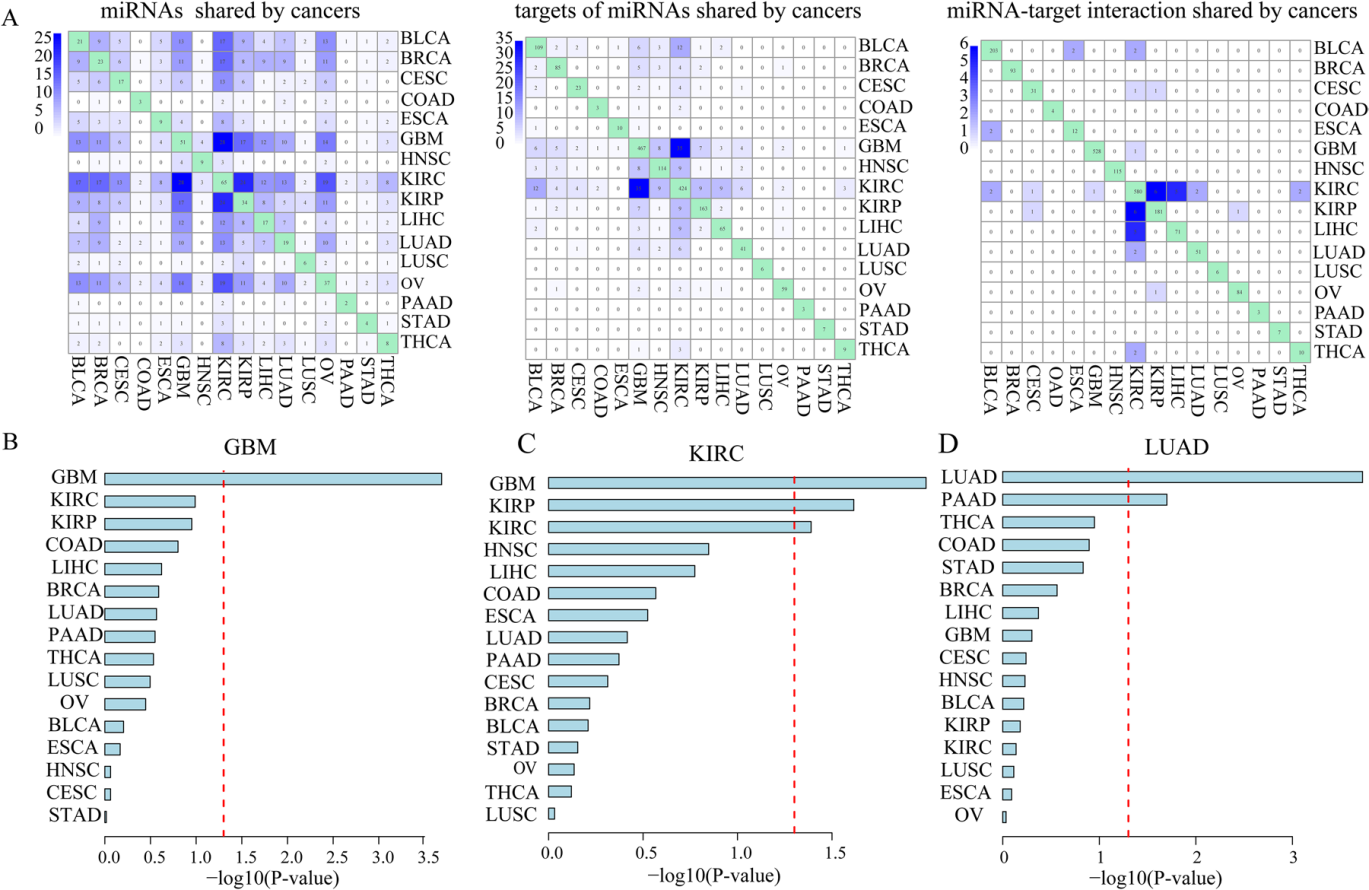
MiRNAs regulated their key targets in a cancer-specific manner **(A).** The number of common miRNAs, key miRNA target genes and key miRNA-target interactions shared among all of 16 cancer types. Bar graph indicates –log 10-transformation *P*-values from multivariable Cox proportional hazards regression models which are used to examine whether key miRNA-target interactions identified in GBM **(B)**, KIRC **(C)** or LIHC **(D)** could be of clinical benefit for other cancer types.

Focusing on prognosis-related key miRNA-targets in 16 cancer types, we examined the global patterns of key miRNAs, target genes and miRNA-target interactions across different cancer types. We found that a small number of key miRNAs were shared by several cancer types. However, we noted that miRNAs tended to regulate distinct key target genes in different cancer types, revealing highly cancer-specific miRNA-target regulation (Figure 5A, Supplementary Table 1, Supplementary Table 2 and Supplementary Table 3). For example, although GBM shared 56.9% (29 out of 51) key miRNAs with KIRC, they had only one common key miRNA-target interaction. The KIRP shared 70.6% (24 out of 34) key miRNAs with KIRC [40], but they had only 6 common key miRNA-target interactions. It indicates distinct underlying pathogenesis for different cancer types and even for two types of kidney cancer, which is consistent with previous observations about KIRC and KIRP [32].

Then, we sought to examine whether key miRNA-target interactions identified in a specific cancer type could be of clinical benefit for other cancer types. We used these key miRNA-target interactions identified in a given cancer type to cluster patients with another cancer type. A multivariable Cox proportional hazards regression model was used to adjust for gender and age. We found that in most TCGA cancer types (11 out of 16; 68.8%) key miRNA-target interactions did not provide prognostic value in more than one other cancer type (Supplementary Figure 5). For example, the combination of key miRNA-target interactions identified in GBM could not have an effect on clinical outcome in any other cancer type (Figure 5B). Besides, the combined key miRNA-target interactions identified in KIRC is significantly associated with survival in patients with KIRP, another type of kidney cancer, and GBM (Figure 5C).

Interestingly, we found that the combination of key miRNA-target interactions identified in malignant adenocarcinomas including LUAD, STAD and COAD could be an independent predictor for overall survival of another adenocarcinoma PAAD patients, individually (Supplementary Figure 5). Notably, key miRNA-target interactions identified in LUAD which originated from lung tissue, could not be an independent predictor for overall survival of LUSC from the same tissue (Figure 5D). It is consistent with a previous study in which transcriptome-based pan-cancer clustering showed that 87.7% of LUSC and 99.7% of HNSC were clustered together, however, LUAD and a subset of BRCA were clustered together [41]. These results suggest that miRNAs contribute to cancer prognosis and metastasis by regulating cancer-specific targets.

### MiRNATarget: a database of prognosis-related key miRNA-target interactions

**Figure 6:**
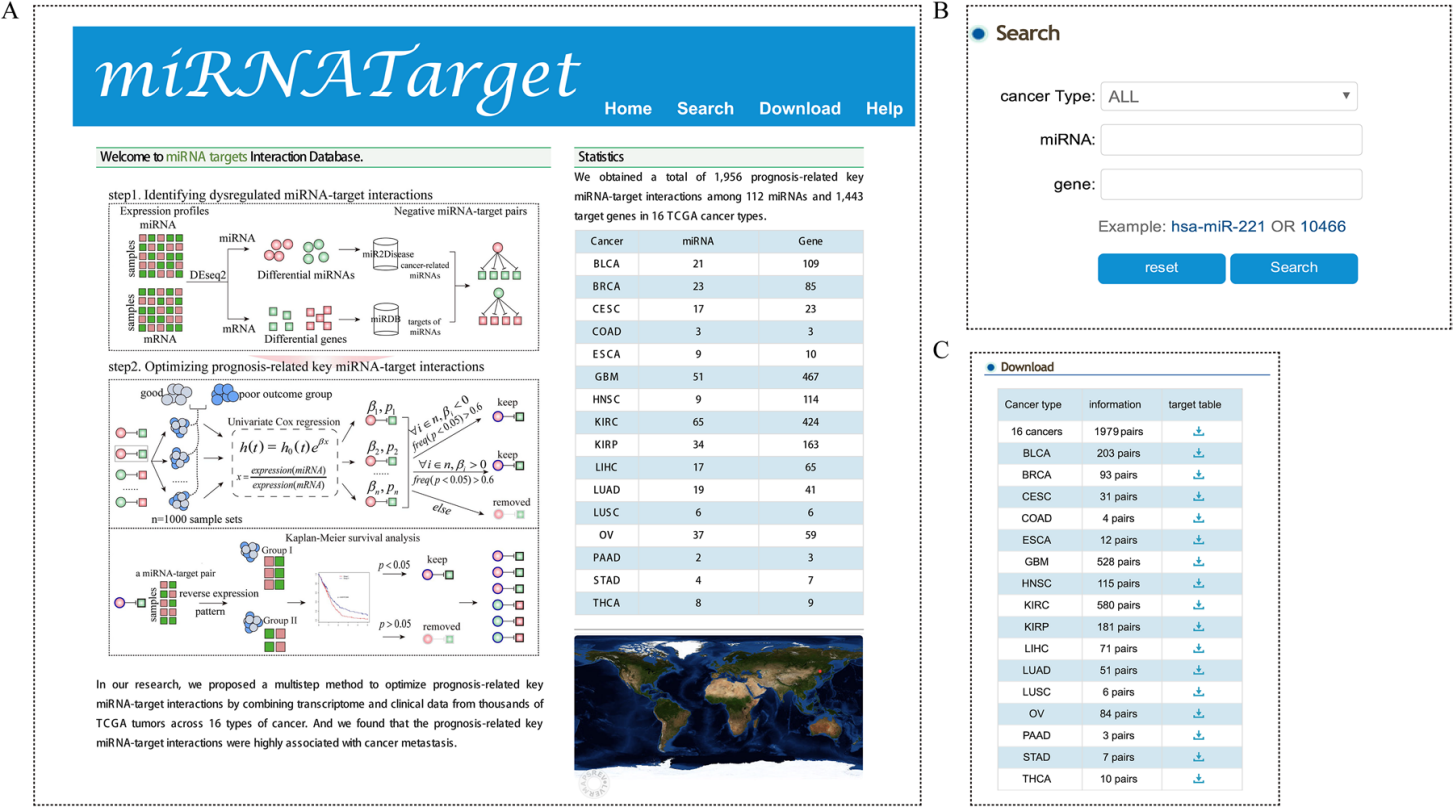
Schematic illustration of the miRNATarget database The home page **(A)**, “Search” module **(B)** and “Download” module **(C)** of the miRNATarget are provided.

We developed a free online database named miRNATarget (http://biocc.hrbmu.edu.cn/miRNATar/) that provides tools for accessing prognosis-related key miRNA-target interactions. These key miRNA-target interactions have a crucial role in cancer metastasis. The database provided a total of 1,956 prognosis-related key miRNA-target interactions involving 112 miRNAs and 1,443 target genes across 16 types of human cancer (Figure 6A). The miRNATarget allows users to retrieve data on the basis of cancer type, miRNA name, or Entrez gene ID of interest, and a report page gives a quick overview of the prognosis-related key miRNA-target interactions, the associated cancer types and Kaplan-Meier survival curves (Figure 6B). Search results can be downloaded as a tab-delimited file (Figure 6C).

## DISCUSSION

MiRNAs prove to be associated with cancer progression and prognosis by regulating key target genes. Identifying prognosis-related key miRNA-target interactions helps to prioritize which genes are their downstream key targets and to further understand the potential molecular mechanisms of how miRNAs are involved in cancer prognosis. In this study, we integrated miRNA and mRNA expression profiles, and clinical information to identify prognosis-related key miRNA-target interactions across 16 different cancer types.

As a result, 1,956 prognosis-related key miRNA-target interactions consisting of 112 miRNAs and 1,443 target genes were identified in 16 types of cancer. To explore the function of prognosis-related key miRNA-target interactions in cancer, GO biological process and hallmark analysis of key miRNA targets were performed. These key target genes are specifically involved in tumor progression-related functions, such as ‘cell adhesion’ and ‘cell migration’. For example, our data demonstrate that miR-21 and miR-155 are related with progression and invasion of GBM by regulating marker genes of cancer metastasis, such as *LHX6*, *DRD1*, *NEUROG1* and *RAB27B*, and thereby contributing to GBM patient survival. A hallmark of metastasis ‘tissue invasion and metastasis’ is significantly influenced by key miRNA targets in ten distinct types of cancer. The results appear to be consistent with the ideas of several studies that cancer metastasis is related to shorter survival and poor prognosis [42–44]. Moreover, by searching the PubMed database, key miRNAs in GBM (or KIRC) were found to be significantly associated with metastasis of GBM (or KIRC) when compared with other disease-associated miRNAs. These results imply that prognosis-related key miRNA-target interactions contribute to cancer metastasis.

By comparing key miRNA-target interactions across multiple cancer types, we observed that key miRNA-target interactions were markedly cancer-type specific, with only a small number of miRNAs shared across several cancer types. This finding highlights that a miRNA may be involved in cancer metastasis of several cancer types, but it works by regulating different targets in different cancer types. Interestingly, we found that key miRNA-target interactions identified in malignant adenocarcinomas could be an independent predictor for overall survival of another adenocarcinoma, indicating their adenocarcinoma cell features [45]. For example, key miRNA-target interactions identified in LUAD are significantly associated with survival in PAAD patients. However, they would not have an effect on clinical outcome in patients with squamous cancer LUSC even though it originated from the same tissue type. In addition, there are more common miRNAs between two types of kidney cancer (KIRC and KIRP) than other cancer types. It supports the common traits behind cancers that some chemotherapeutic drugs are able to treat several types of cancer, such as cisplatin [46]. However, there are very few common key miRNA-target interactions between KIRC and KIRP, indicating distinct underlying pathogenesis for these two types of kidney cancer [32]. More importantly, cancer type-specific key miRNA-target signatures may provide a robust approach towards personalized medicine in cancer prognosis and treatment and reduce the incidence of side effects.

In summary, we present a computational approach for optimizing prognosis-related key miRNA-target interactions by combining large numbers of mRNA and miRNA expression profiles and clinical information from 5,353 patients across 16 TCGA cancer types. The results highlighted that prognosis-related key miRNA-target interactions were highly associated with cancer metastasis. We provided a free online database named miRNATarget for optimizing prognosis-related key miRNA-target interactions across 16 types of cancer.

## MATERIALS AND METHODS

### Data sources

MiRNA-seq (n = 6046), RNA-seq (n = 5672) and clinical data (n=5042) of 14 cancer types, including bladder urothelial carcinoma (BLCA), breast invasive carcinoma (BRCA), cervical squamous cell carcinoma and endocervical adenocarcinoma (CESC), colon adenocarcinoma (COAD), esophageal carcinoma (ESCA), head and neck squamous cell carcinoma (HNSC), kidney renal clear cell carcinoma (KIRC), kidney renal papillary cell carcinoma (KIRP), liver hepatocellular carcinoma (LIHC), lung adenocarcinoma (LUAD), lung squamous cell carcinoma (LUSC), pancreatic adenocarcinoma (PAAD), stomach adenocarcinoma (STAD) and thyroid carcinoma (THCA), were downloaded from The Cancer Genome Atlas project (TCGA) (Table 1). For glioblastoma multiforme (GBM) and ovarian serous cystadenocarcinoma (OV), the mRNA and miRNA microarray data and clinical information were obtained from the TCGA project. Detailed sample information is described in Table 1.

### Identifying differentially expressed genes and miRNAs

For sequencing data (RNA-seq and miRNA-seq), genes with at least 10 reads in more than 50% samples and miRNAs with at least 2 reads in more than 50% samples were retained for further analyses. The log-transformed RPKM (read per kilobase of exon per million mapped reads) and log-transformed RPM values (reads per million miRNA mapped) were used to calculate the mRNA and miRNA levels within each cancer type, respectively. Differentially expressed miRNAs and mRNAs were identified using DESeq2 [47] (FDR<0.05, fold change>1.2). For microarray data, genes or miRNAs with missing values in more than 30% sample were removed from the analysis. Microarray data processing and normalization utilized Robust Multiarray Analysis (RMA) and quantile normalization with the Bioconductor package Affy. Differential expression relative to matched controls was performed with the significance analysis of microarrays (SAM) algorithm using Bioconductor Siggenes package (FDR<0.05, fold change>1.2). For each type of cancer, a gene (or miRNA) was considered to be high- or low-expressed relative to being above or below the median expression value across cancer samples.

### Identifying prognosis-related key miRNA-target interactions

#### Identifying dysregulated miRNA-target interactions

Cancer-related miRNAs were obtained from miR2Disease database [48]. The predicted targets of miRNAs were derived from miRDB database [49]. For each cancer type, the dysregulated miRNA-target interactions were identified by several criteria: (1) cancer-related miRNAs and their targets were differentially expressed, (2) the direction of differential expression between miRNAs and their targets was reversed, (3) the pattern of a high-expressed miRNA regulating its low-expressed targets (or vice versa) was present in more than 20% cancer samples. A set of 50,805 miRNA-target interactions between 131 miRNAs and 11,396 genes was obtained.

#### Optimizing prognosis-related key miRNA-target interactions

To evaluate whether the dysregulated miRNA-target interactions were associated with prognosis, we selected 5,353 patients from 16 cancer types who had expression of miRNAs and mRNAs and survival data (Table 1). For each type of cancer, the upper and lower bound of 95% confidence interval (CI) for the median survival time was estimated. Patients whose survival time was above (below) the upper (lower) CI were categorized into the good (poor) outcome group. Next, we generated 1,000 sample sets for survival analysis by randomly selecting 80% samples from the poor outcome and good outcome group, respectively. We performed univariate Cox regression model according to expression ratio of a miRNA to its target to evaluate the influence of the miRNA-target interaction on overall survival (OS) in each of the above sample sets individually. A miRNA-target interaction was selected under the condition that it’s regression coefficient (β) was required to be positive (or negative) in each univariate Cox regression analysis and the frequency of *P*-value<0.05 was greater than 0.6. For a miRNA-target interaction, patients with specific cancer types were divided into two groups on the basis of reverse expression pattern of the miRNA and its target. The first group consisted of patients with a high-expressed miRNA regulating its low-expressed target and the second group of patients with a low-expressed miRNA regulating its high-expressed target. The survival difference between the two groups was assessed by the Kaplan–Meier analysis and *P*-value was determined using log-rank test. MiRNA-target interactions with log-rank test *P*-value<0.05 were considered as prognosis-related key miRNA-target interactions.

### Identifying hallmarks of cancer affected by prognosis-related key miRNA-target interactions

GO Terms associated with the hallmarks of cancer were obtained from [50, 51]. Human protein-protein interaction network (PPI) is obtained from HPRD. Human KEGG pathways from Synapse (syn1741407) which shows the semantic similarity score >0.3 with a hallmark-associated GO term using R package ‘GOSemSim’, are considered to be associated with hallmarks of cancer. For each hallmark-associated KEGG pathway, random-walk analysis of the protein interaction network with a restart probability of 0.7 [52] was performed to measure long-range correlations between the KEGG pathway and prognosis-related key targets of miRNAs (seed gene set). The probabilities of genes in the PPI network under the steady state were obtained, which characterized the influence of key miRNA targets on genes in hallmark-associated KEGG pathways. For each hallmark of cancer, the median of probabilities was considered to be a score to measure the impact of prognosis-related key miRNA-target interactions on the cancer hallmark. To investigate the significance of impact of prognosis-related key miRNA-target interactions on a specific hallmark of cancer, we perturbed the PPI network for 1,000 times by rewiring every edge (keeping the degree distribution of the original network). Based on permutation test, the *P*-value was calculated as the fraction of permutations that lead to a greater than or equal number of random scores than those observed scores.

## SUPPLEMENTARY MATERIALS FIGURES AND TABLES




