# Supplementary material for: Optimizing prognosis-related key miRNA-target interactions responsible for cancer metastasis
Source: Oncotarget. 2017 Nov 27;8(65):109522–35. doi: 10.18632/oncotarget.22724 (PMC5752539; doi:10.18632/oncotarget.22724)
Supplement: Supplementary file 1 [file oncotarget-08-109522-s001.pdf]

Optimizing prognosis-related key miRNA-target interactions responsible for cancer metastasis

SUPPLEMENTARY MATERIALS

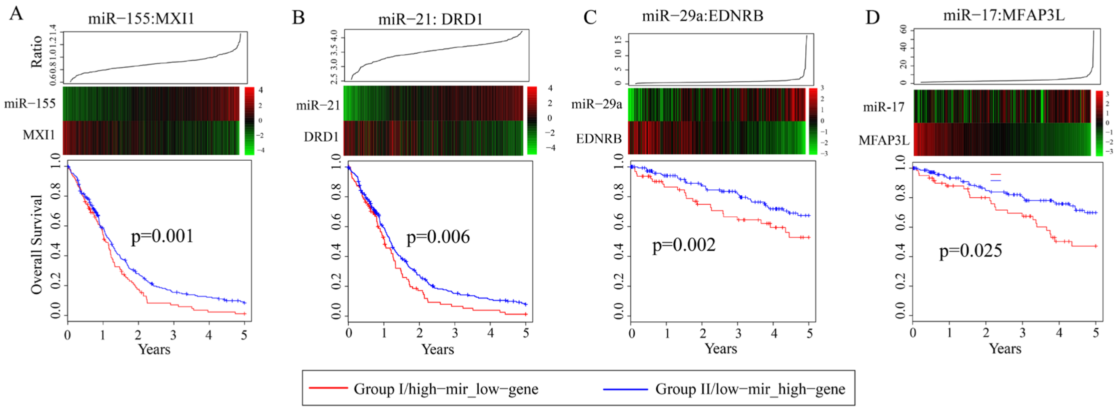

Supplementary Figure 1: Survival analysis of patients grouped using the expression levels of miRNAs and their key target genes. The distribution of expression ratio of a miRNA to its key target gene (top). Heatmap of the expression of a miRNA and its key target gene (middle). K-means clustering of patients with GBM was performed according to the expression ratio of miR-155 to *MXI1* **(A)** or miR-21 to *DRD1* **(B)**. K-means clustering of patients with KIRC was performed according to the expression ratio of miR-29a to *EDNRB* **(C)** or miR-17 to *MFAP3L* **(D).** A corresponding Kaplan-Meier survival plot of the two groups of patients identified by reverse expression pattern of the miRNA to its target was constructed (bottom).

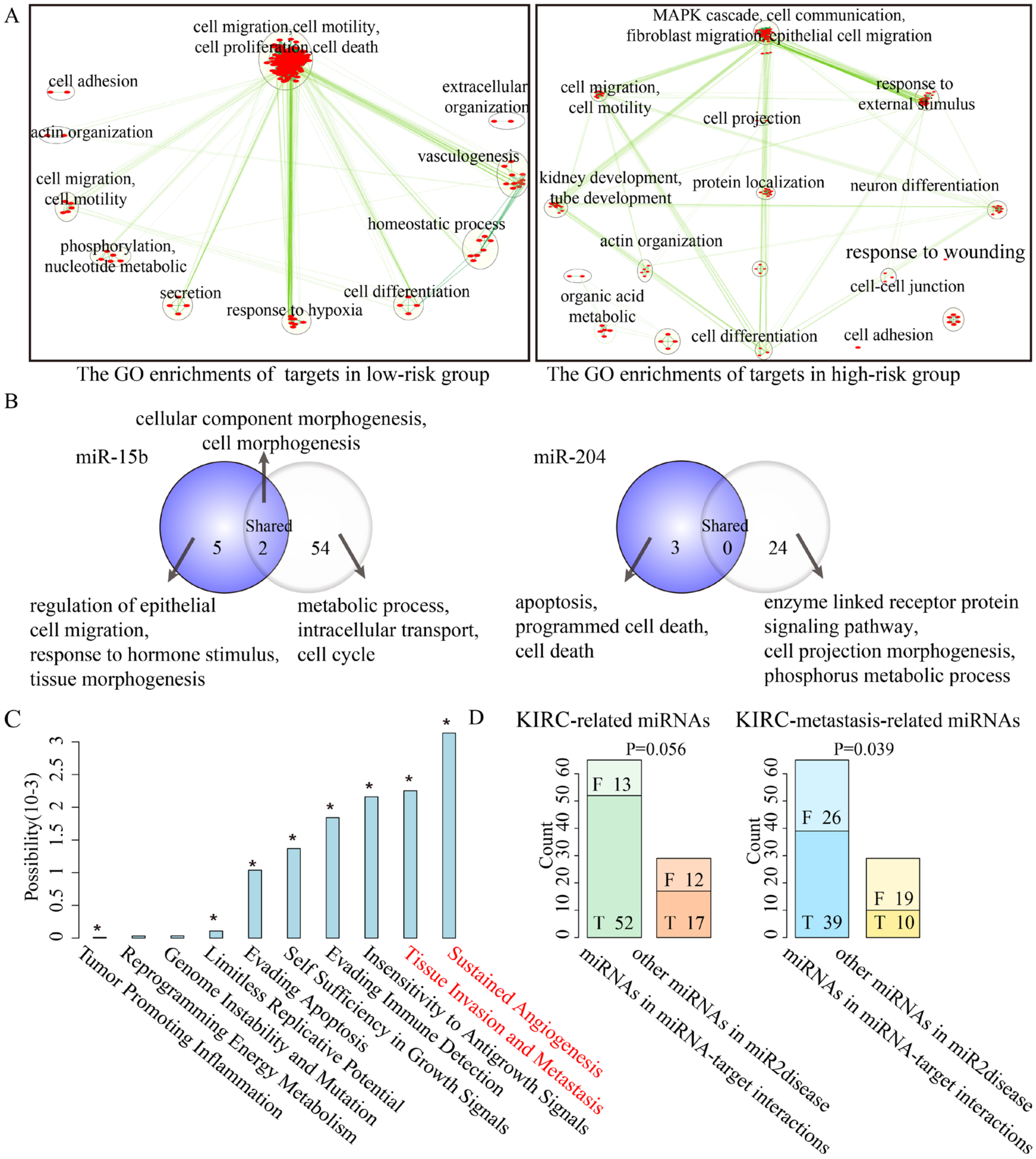

Supplementary Figure 2: The function explorations of prognosis-related key miRNA-target interactions in KIRC. (A). Map of enriched functions for genes in prognosis-related key miRNA-target interactions based on DAVID output. **(B).** Overlap between the DAVID output detected by the key targets (left, purple) and all targets (right) of miR-29a and miR-17 in KIRC. The labels of top most significant GO terms are showed. **(C).** The impact of prognosis-related key miRNA-target interactions on 10 hallmarks of cancer. Asterisks represent significant levels at *P*-value<0.05 based on permutation tests. **(D).** Bar graphs showing the number of selected miRNAs and other cancer-related miRNAs classified into KIRC-related miRNAs (left panel), KIRC metastasis-related miRNAs (right panel) or not, respectively.

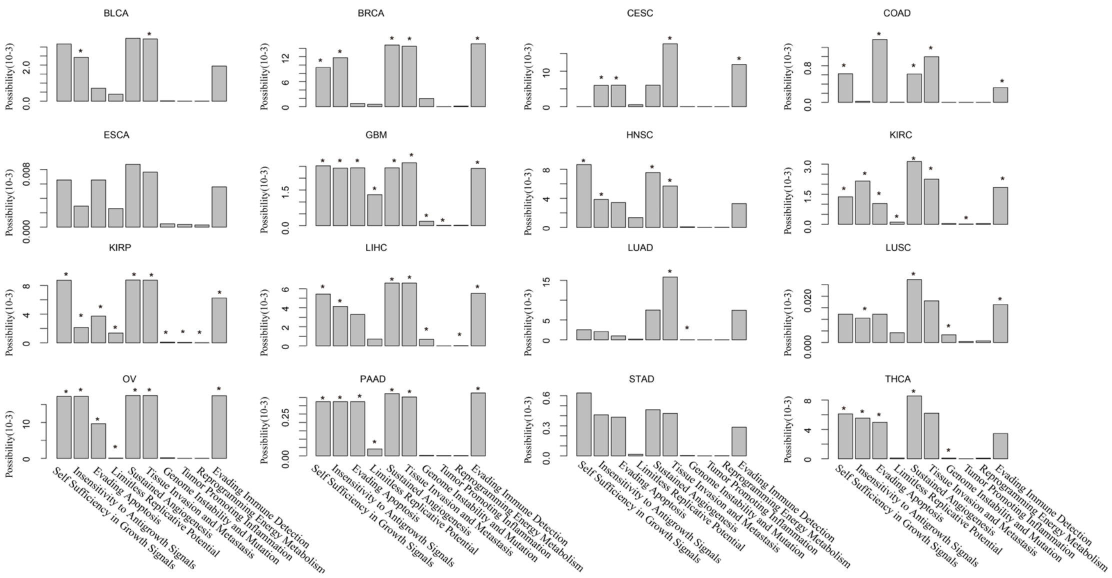

Supplementary Figure 3: The function explorations of prognosis-related key miRNA-target interactions selected from KIRC. The impact of prognosis-related key miRNA-target interactions on 10 hallmarks of cancer in each type of cancer. Asterisks represent significant levels at *P*-value<0.05 based on permutation tests.

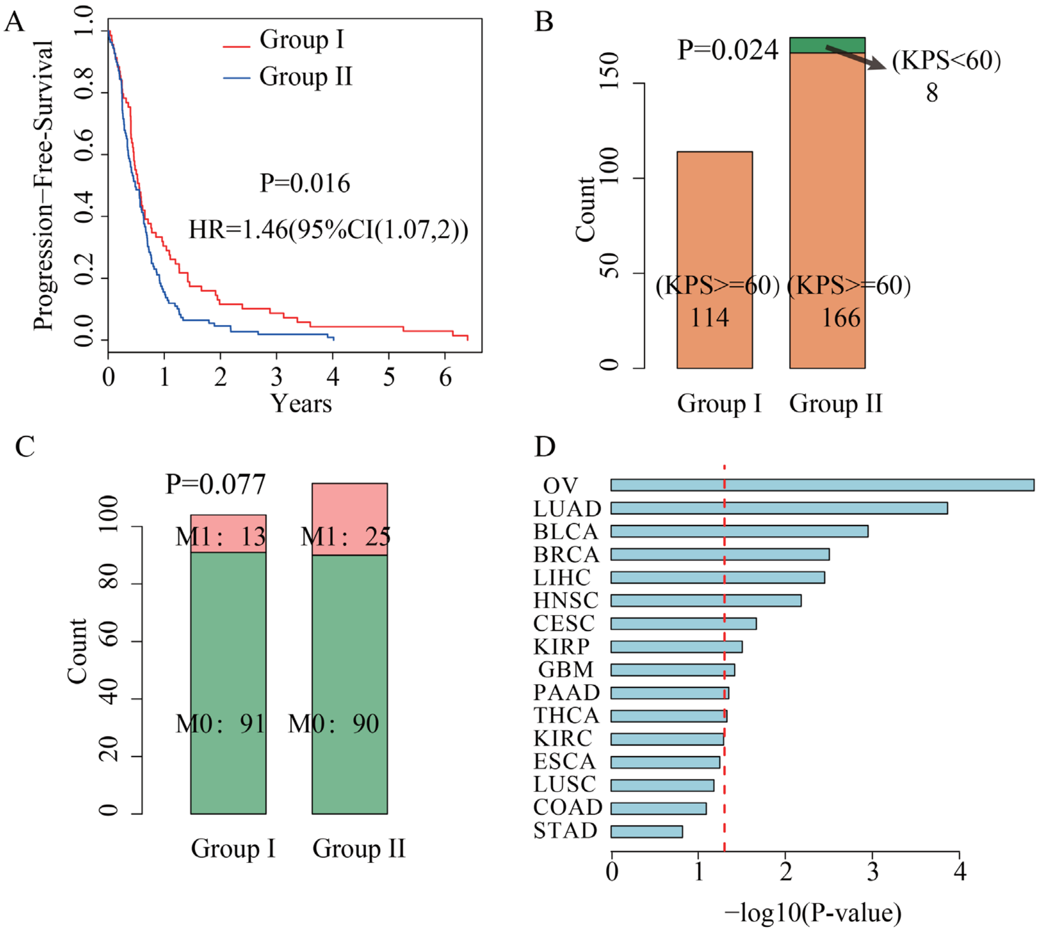

Supplementary Figure 4: Clinical significance of the combination of key miRNA-target interactions for 16 human cancers. **(A).** Kaplan-Meier estimates of the progression-free-survival using the key miRNA-target interactions in GBM. Bar graphs showing the number of GBM patients with KPS<60 or not **(B)** and with (M1) or without (M0) distant metastases **(C)**. **(D)** For each type of cancer, bar graph inicates –log 10-transformation of *P*-values from multivariable Cox proportional hazards regression analysis using age, gender and key miRNA-target interactions identified in the corresponding cancer type.

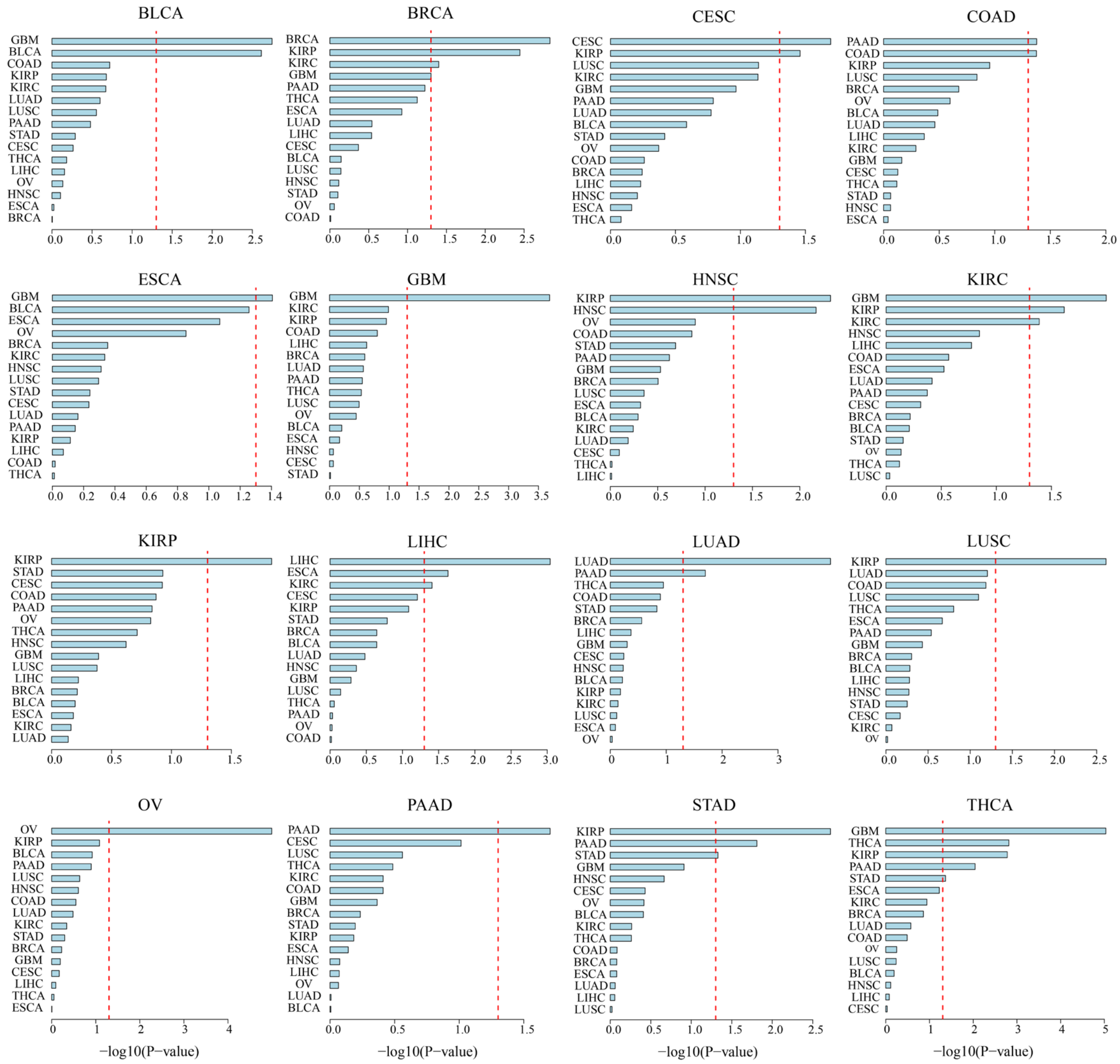

Supplementary Figure 5: MiRNAs regulated their key targets in a cancer-specific manner. Bar graph indicates –log 10-transformation of *P*-values from multivariable Cox proportional hazards regression analysis using age, gender and key miRNA-target interactions identified in a given cancer type.

Supplementary Table 1: The miRNAs shared among 16 cancer types

See Supplementary File 1

Supplementary Table 2: The key target genes of miRNAs shared among 16 cancer types

See Supplementary File 1

Supplementary Table 3: The key miRNA-target interactions shared among 16 cancer types

See Supplementary File 1
